# Manual Physical Therapy for Non-Surgical Treatment of Adhesion-Related Small Bowel Obstructions: Two Case Reports 

**DOI:** 10.3390/jcm2010001

**Published:** 2013-02-04

**Authors:** Amanda D. Rice, Richard King, Evette D’Avy Reed, Kimberley Patterson, Belinda F. Wurn, Lawrence J. Wurn

**Affiliations:** Clear Passage Physical Therapy, 4421 NW 39th Ave, Ste 2-2, Gainesville, FL 32606, USA; E-Mails: amandar@clearpassage.com (A.D.R.); richardk@clearpassage.com (R.K.); evetter@clearpassage.com (E.D.R.); kimp@clearpassage.com (K.P.); belindaw@clearpassage.com (B.F.W.)

**Keywords:** small bowel obstruction, SBO treatment, manual physical therapy, surgery alternative

## Abstract

Background: Adhesion formation is a widely acknowledged risk following abdominal or pelvic surgery. Adhesions in the abdomen or pelvis can cause or contribute to partial or total small bowel obstruction (SBO). These adhesions deter or prevent the passage of nutrients through the digestive tract, and may bind the bowel to the peritoneum, or other organs. Small bowel obstructions can quickly become life-threatening, requiring immediate surgery to resect the bowel, or lyse any adhesions the surgeon can safely access. Bowel repair is an invasive surgery, with risks including bowel rupture, infection, and peritonitis. An additional risk includes the formation of new adhesions during the healing process, creating the potential for subsequent adhesiolysis or SBO surgeries. Objective: Report the use of manual soft tissue physical therapy for the reversal of adhesion-related partial SBOs, and create an initial inquiry into the possibility of nonsurgical lysis of adhesions. Case Reports: Two patients presenting with SBO symptoms due to abdominal adhesions secondary to abdominal and pelvic surgery were treated with manual soft tissue physical therapy focused on decreasing adhesions. Conclusions: Successful treatment with resolution of symptom presentation of partial SBO and sustained results were observed in both patients treated.

## 1. Introduction

Peritoneal adhesions are a major contributory factor in the development of small bowel obstruction (SBO) worldwide and are a common post-abdominopelvic surgical occurrence. The literature indicates that 50% to 100% of patients develop adhesions after any abdominal/pelvic surgery [1,2,3,4,5]. A large analysis of the incidence of adhesions which was performed in the 1990’s found that 35% of all open abdominal or pelvic surgery patients were readmitted to the hospital more than twice to treat post-surgical adhesions within 10 years following their initial surgery, with 22% of readmissions requiring surgery occurring within the first year. The report noted “readmissions continued steadily throughout the 10-year period” of the study [6].

The severity of post-surgical adhesions are challenging to control, as they are influenced by factors attributed to both the patient and surgical procedure. Despite numerous clinical trials to assess the validity of various strategies, agents and surgical meshes to prevent adhesion formation, the debate continues with no protocol yet shown that prevents the generation of adhesions as the body heals from surgical intervention [2].

Adhesions commonly form secondary to the normal wound healing process. They form as a result of fibrin formation at the site of the surgical wound which provides the matrix for fibroblasts to migrate and generate a collagen extracellular matrix (ECM) [2]. It is this ECM and its associated cells that ultimately become the adhesions. Although it is the body’s response to surgery and the healing process in the presence of increased levels of inflammatory cytokines that promotes the formation of adhesions, it is clear that adhesions are not merely nonfunctional scar tissue. It has been found that mature adhesions are complex tissues with ingrowths of capillaries, adipose tissue, smooth muscle and nerve fibers that are typically found in complex regenerating structures [7]. Further, adhesion formation is not always limited to the geography of the locally traumatized tissues. Some patients form additional adhesions at locations proximal or distal to the surgical site. It should be noted that surgery is not the only cause of adhesion formation and that all healing, including surgery, physical trauma, infection and/or inflammation has the ability to form adhesions in the body [8].

The standard current treatment for SBO due to adhesions that cannot be resolved by gastric decompression or bowel rest is surgery to lyse the adhesions (adhesiolysis), thereby resolving the obstruction. In some cases, SBO surgery also involves cutting and removing adhered, damaged or necrosed sections of the bowel, followed by resection of the remaining bowel ends. Utilizing surgery to lyse the adhesions caused by an initial surgery and/or to resect the bowel often causes the formation of new adhesions as a part of the wound healing process. This can create and perpetuate an ongoing cycle of healing and adhesion formation, obstruction surgery to remove the offending adhesions or resect the bowel, followed by new healing and new adhesion formation, and so on. In cases of SBO, this cycle can mean repeat hospitalizations to treat a life-threatening condition. The risk of infection due to spillage of bowel contents into the interstitial spaces of the abdomen is not an insignificant complication following bowel resection. Thus, any method that can be used to slow, prevent, or non-surgically release post-surgical adhesions that compromise the bowel is of significant value to physicians and their patients.

Adhesions have also been identified as a leading cause of secondary female infertility [9]. The Clear Passage Physical Therapy group has previously reported the successful use of this manual soft tissue physical therapy in the treatment of fallopian tube occlusion and treatment of dyspareunia and dysmenorrhea secondary to adhesions [10,11]. Following publication of those studies, the same manual soft tissue physical therapy was applied to patients with documented abdominal adhesions following recurrent abdominal surgeries, and to patients presenting with current or recurring partial and total SBOs as a non-surgical treatment option.

## 2. Experimental Section

The two patients presented in this retrospective study were required to complete a standard Patient Intake Questionnaire detailing their pain, medical and surgical history. Body Mass Index was calculated for each patient using the online BMI calculator from the NIH National Heart Lung and Blood Institute. [12] 

Those patients who indicated a history of small bowel obstruction (SBO) on the patient intake form were asked if they were willing to allow us to use their data in a small, investigative, retrospective study. Each patient then signed an Informed Consent document. Frequency, timing and severity of SBO episodes and all abdominal or pelvic surgeries were noted in their charts. Each patient was required to submit all relevant medical records and operative reports from which the medical history presented in this case study is summarized. Both patients presented in this case report had sought medical treatment and diagnostics for adhesions and bowel obstructions from multiple physicians and radiologists prior to manual physical therapy treatment.

Previous medical histories for each patient were obtained in accordance with HIPPA regulations. Detailed clinical records were kept of each patient throughout the course of therapy including symptomatic complaints, areas treated, techniques used, and treatment date/duration in accordance with the American Physical Therapy Association guidelines.

Upon initial evaluation and discharge assessment, patients were asked to rate their pain on a scale from 0 to 10, with 0 being no pain and 10 being debilitating pain. The patients were also asked about quality of life measures including impact of pain on daily activities and dietary restrictions due to their condition.

Findings from the initial evaluation, which included patient history, previous pathology reports and physician diagnoses, and visual, palpatory, postural and movement exams were correlated to determine dysfunctional areas needing treatment. In these cases, restrictions, scars or adhesions were palpated by the therapists, major organs were identified, and decreased mobility of specific organs was determined. The evaluation included layer palpation of the myofascial and visceral structures; starting with the most superficial structures and progressing to the deepest. The development of tactile skills includes the ability to detect tissue and visceral texture abnormalities and restricted mobility [13]. The therapists assessed tissue temperature, moisture, shear, extensibility and texture, throughout the abdominopelvic viscera, with a special focus on areas deep to surgical scars. Testing visceral mobility consisted of the therapist palpating the tissues, then making precise manual movements to assess the ability of the organs to move directly over surrounding organs and tissues. 

The patients were treated via a site-specific intensive manual physical therapy that occurred for 4 h a day for 5 consecutive days. In the case of these two patients, approximately 70% of the therapists’ time was utilized directly on the adhered areas of the abdomen and pelvis. The manual physical therapy protocols utilized by the therapists have been previously described in other studies where the primary focus of therapy was the manual decrease of adhesions, and the outcomes were a return of normal mobility and motility to previously adhered organs. [10,11,14] Thus, the therapy described in these studies and on those patients may be applied to adhered areas of any of the abdominal or pelvic organs, including the reproductive, digestive, urinary and other organs within and external to the peritoneum.

Briefly, the protocol consists of over 200 individual manual techniques focused on creating what we theorize are micro-failure of the adhesive crosslinks, the building blocks of adhesions, by application of various site-specific pressures across adhered areas of the abdominal viscera. Because no means of visual confirmation exists (excluding surgery, which was not performed), macro-failure or deformation of adhesions could only be assumed as visceral mobility improved, and as patients reported increased function and decreased pain. In earlier published studies, the manual physical therapy techniques used for treating abdominal adhesions in patients with histories of adhesion related SBO were used to clear adhesion related blocked fallopian tubes. Independent blinded radiologic tests documented that the patient’s fallopian tubes were no longer blocked by adhesions post treatment.

The amount and duration of force that is applied to cause these microfailures can be significant, but varies within the tolerance of each patient and according to the site of the body that is being treated. For example, the same technique applied to the abdomen will use much greater force than when applied to the ovary or fallopian tubes. The manual physical therapy approach is one of whole body treatment, treating not only the abdomen in these patients, but also other areas of the body in which decreased function or mobility was identified during the evaluation. In general, patients received about 14 h of this manual therapy directly to perceived adhered abdominal structures for every 20 h of therapy; the remaining six hours were spent with history review, evaluation, treating other symptomatic areas including nearby myofascial structures (low back, lower extremities, shoulders and neck, *etc.*), and paperwork. Via palpation and an understanding of the anatomy, therapists focused on treating the adhesions, rather than treating the underlying viscera. Except at their ligamentous attachments and their normal anatomical attachment to other organs, abdominal and pelvic organs should be able to glide freely over each other. When therapists were unable to palpate freely gliding organs, the restrictions were noted as decreased mobility. This frequently occurred at the sites of prior surgeries.

While the force used by the therapists was sometimes significant, it is interesting to note that except for some temporary soreness, there have never been any significant adverse events in the hundreds of patients treated with abdominal or pelvic adhesions, using this therapy. This observation seems to refute the commonly held belief among some manual therapists that abdominal structures should only be treated lightly, and infrequently. Notwithstanding, a BMI of above 36 and surgery within 12 weeks preceding therapy are considered contraindications by the clinic for this treatment. Both patients presented in this case report had low BMIs allowing for easy palpation of the internal organs and adhesions, scars and restrictions. In clinical trials and published studies, these techniques increased mobility and decreased pain in patients with significant surgical histories indicating adhesion formation [10,14,15,16].

Patients are typically followed for at least a year following treatment via voluntary survey.

## 3. Case Reports

### 3.1. Patient 1

**Figure 1:**
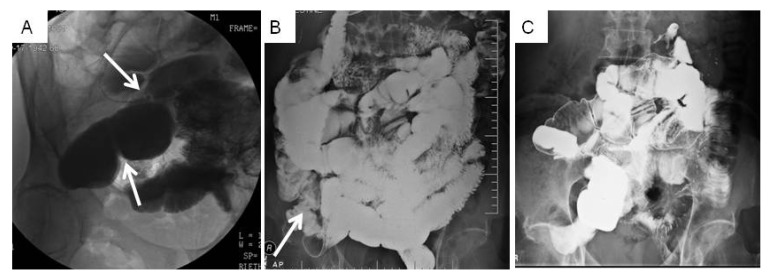
Small bowel radiographs of Patient 1 documenting SBO resolution over time. Arrows note areas of obstruction. (**A**) Before therapy in 2011: incomplete SBO due to adhesions visualized by X-ray showing dilation of the proximal mid ileum. (**B**) Twelve months after therapy in 2012: mild stricture at the terminal ileum with no other small bowel abnormalities. (**C**) After 40 h of therapy: normal small bowel series X-ray in 2012.

Patient 1 was a 69 year old Caucasian male with a BMI of 17.7 and a history of GERD, asthma, hypertension, emphysema, recurrent SBO and bilateral inguinal hernias. His surgical history included SBO surgery at birth, childhood appendectomy, 3 laparoscopically repaired hernias from 2008–2010 with a surgical mesh inserted, Nissen fundoplication and partial thyroidectomy in 2010, laparotomy for SBO secondary to extensive adhesions in July 2010 and January 2011. Immediately after surgery in 2011, Patient 1 underwent a small bowel radiographic series that showed an incomplete SBO at the junction of the proximal mid ileum, and dilation throughout the small bowel, likely due to adhesions per the radiologist (Figure 1A). He was still experiencing symptoms of SBO (bloating, nausea, inability to eat normally) three months following the 2011 laparotomy, and reported a significant impact on his quality of life, focused on the changes to his diet that occurred after the SBO. He also reported abdominal tightness and significant intermittent pain (10/10 on a pain scale).

Patient 1 received no further medical interventions after his 2011 radiography prior to receiving this manual physical therapy treatment. Upon initial physical therapy evaluation, he presented with pain; visible scarring superficial to tissues that felt like deep palpable adhesions (consistent with his physician’s 2011 diagnosis of abdominal scarring), decreased visceral mobility (decreased ability for organs to glide normally), and tightness throughout the abdomen; myofascial, osseous and visceral hypomobility; and decreased quality of life due to SBO. He also presented with decreased strength and range of motion in both hips and trunk, and in his cervical, thoracic, and lumbar spine at the initiation of his physical therapy regimen. 

In 2011, Patient 1 underwent 20 h of intensive manual physical therapy over the course of five days with a focus on improving range of motion, and decreasing scar tissue, adhesions, and soft tissue restrictions. Primary goals of therapy included decreased SBO symptoms and a decrease of future bowel obstructions. At discharge from physical therapy, Patient 1 presented with improved range of motion in trunk, cervical, thoracic, and lumbar spine, and hips by at least 5 degrees for each range of motion measurement (data not shown). Abdominal mobility and tightness had also improved. Upon return to his home state, he underwent follow-up diagnostic evaluations by a blinded, independent radiologist. Findings showed an ileum stricture but no other obstructions in the bowels (Figure 1B). Furthermore, this patient reported no further obstructions or SBO symptoms over the 12 months following therapy.

In 2012, following a diagnostic test showing a decreased diameter of the ileum (Figure 1B), Patient 1 was treated again for 20 h over the course of 5 days. Once again, he demonstrated improvement in all areas tested after twenty additional hours of the manual physical therapy including a minimum of 5 degrees increase in all ranges of motion that were measured to be decreased at the re-evaluation (data not shown). Ten days after the conclusion of therapy, the patient underwent a small bowel series, again conducted by a blinded independent radiologist. Results showed a normal bowel, with no kinking, constricting lesions or abnormal masses noted (Figure 1C). Patient 1 reported no further SBO symptoms for one year following the second course of treatment, for a total time of two years with no further medical interventions for SBO or SBO symptoms.

### 3.2. Patient 2

Patient 2 was a 49 year old Caucasian female with a BMI of 21.8 and a significant history of recurrent abdominal adhesions as a result of multiple surgeries and injuries including benign lumpectomy of the breast, dilation and curettage, motor vehicle accident with injury to the soft tissue of the chest wall resulting in migraine headaches and fibromyalgia, and injuries to bilateral wrists. She also had a past medical history of systemic lupus, chronic fatigue, fractured right upper extremity and several fingers, left knee surgery, mild arthritis, brown recluse spider bites and multiple miscarriages. 

Patient 2 underwent the first of a series of surgeries beginning with a breast lumpectomy. Within weeks of this surgery, she was hospitalized as she was unable to keep any food or fluids down and all bowel movements ceased, accompanied by severe bloating and abdominal pain. After six days in the hospital, an emergency exploratory laparotomy revealed adhesion-related bowel obstructions. She had cysts removed from her ovaries and colon, as well as an appendectomy in June 2006. Two weeks later (July 2006) she underwent her second abdominopelvic surgery, the first to treat adhesion-related small bowel obstruction, in which the surgeon resected a fibrous band from her transverse colon to her posterior peritoneum, which had caused an internal herniation. In August 2006, the patient underwent a third abdominopelvic surgery, an exploratory laparotomy for a spigelian hernia, ovarian mass resection, oophorectomy, and adhesiolysis. 

Despite undergoing two adhesiolysis procedures, the patient continued to experience symptoms of bowel obstruction including abdominal distention, nausea, vomiting, pain and constipation immediately following the surgeries. 

In January 2007, she was again diagnosed with abdominal adhesions, a diagnosis confirmed during a laparotomy performed in July 2007, which was her fourth abdominopelvic surgery. That surgeon noted and lysed dense intra-abdominal adhesions and scar tissue that were binding the loops of the small intestine; the physician also performed an umbilical hernia repair with mesh. In October 2007 she underwent a fifth abdominopelvic surgery: bilateral inguinal hernia repair.

In July of 2008, she underwent a sixth abdominopelvic surgery, another exploratory laparotomy, in which the surgeons noted extensive abdominal adhesions, a nearly complete SBO due to adhesions causing eight to ten acute angle kinks of the bowel and adhesions around the stomach causing it to become elongated, J-shaped and adhered to the anterior abdominal wall. Shortly thereafter, she underwent a complete hysterectomy and oophorectomy due to uterine fibroids and ovarian disease, her seventh abdominopelvic surgery. Thus, she underwent seven abdominopelvic surgeries, most related to adhesions and/or SBO, within a period of 30 months. In November 2008 the patient was diagnosed with recurrent idiopathic abdominal adhesions and informed of the need for an eighth abdominopelvic surgery, a laparotomy for adhesiolysis to treat the recurring SBO symptoms. Feeling that she needed to take another path, she elected to try a manual physical therapy in November 2008 rather than undergo further surgical intervention for her current partial SBO.

During the physical therapy initial evaluation, Patient 2 reported constant dull aching abdominopelvic pain averaging 7/10 on the pain scale; intermittent dull aching pain in her torso at 10/10 on the pain scale; and pain in one lower leg that was intermittent dull aching, averaging 5/10 on the pain scale in an area that was bitten by a brown recluse spider. She reported pain with bowel movements, urination and intercourse. She had lost 18 pounds and was on a totally liquid diet at the time of the initial evaluation. She reported pain and a severe decrease in quality of life, beginning with her first surgery (June 2006) that forced her to leave the workforce. She presented with severe abdominal pain; decreased range of motion at the cervical, thoracic, lumbar and sacral regions. Physical therapy palpation and tests revealed severe visible and palpable scars and adhesions, severe restrictions in visceral abdominal mobility; decreased myofascial and osseous mobility and range of motion; and significant postural asymmetries. Current medication at the initiation of treatment was Darvocet-N 100 every 6 h for pain. 

Like Patient 1, Patient 2 underwent 20 h of intensive manual physical therapy over the course of 5 days with the goals of improving range of motion, flexibility, strength and posture, as well as decreasing adhesions in the bowel and abdominal wall in an attempt to decrease or negate the need for her pending surgery and to prevent a total small bowel obstruction. Again, the therapists used similar techniques as noted in their earlier published studies in which they focused on adhesions affecting endometrial and tubal structures. That is, they treated the adhesions that existed on and within the interstitial spaces, as well as on and within abdominal and pelvic organs, as the primary focus of their therapy.

At discharge from physical therapy, Patient 2 reported that pain had decreased by 90% or more, and she was able to return to a normal diet. The therapist noted improvement in range of motion to within functional limits and improved posture, increased visceral mobility, increased myofascial and osseous mobility, and significantly decreased pain with urination, sexual intercourse and bowel movements. Several weeks following discharge from therapy, Patient 2 reported that she had re-gained the 18 pounds she had lost due to her compromised bowel, and she had returned to a normal quality of life. She was able to cancel the scheduled surgery for adhesiolysis and SBO. At her one-year follow-up from therapy, Patient 2 reported experiencing no additional SBOs or SBO symptoms, and that no further surgical interventions were needed or performed.

## 4. Conclusions

In cases where non-surgical management is unsuccessful, the current standard medical treatment for SBO caused by adhesions is surgery. Unfortunately, surgery is implicated as the most common cause of abdominal adhesions and adhesion-related SBO [5]. Previous studies have shown the decrease in pain associated with abdominal adhesions after surgical adhesiolysis lasted up to one year; the subsequent return of the pain has been hypothesized as due to new, or expanded adhesion formation [17,18,19]. In the US in 2010, 70,194 patients underwent small bowel resection surgery with an average hospitalization of 14.2 days, an average cost of $114,175, and a patient death rate of 6.75%; 89,222 patients underwent surgery for adhesiolysis, an average hospitalization of 8.4 days, an average cost of $65,955, and a patient death rate of 2.3% [20]. Those numbers are staggering when it is considered that, based on prior studies, 35% of those patients that undergo pelvic or abdominal surgery surgery will likely be readmitted for surgical intervention for adhesiolysis over the next 10 years [6].

Here we reported the use of a manual physical therapy for the treatment of SBO symptoms in two patients who presented with numerous, frequently recurring bowel obstructions. Furthermore, both patients had entered a cycle of surgery to remove adhesions, followed by the formation of post-surgical adhesions, followed by another adhesiolysis surgery, and so on. In each case, post-surgical adhesion formation occurred more rapidly and more severely with each successive surgery. Patient 2 experienced only 12 weeks between the most recent surgery for SBO and her next partial SBO, when she received her physician’s recommendation for another (eighth) abdominal surgery. Her surgeon had scheduled the next surgery for adhesiolysis when she opted to try this manual physical therapy treatment instead. 

Both patients reported lasting pain relief and avoidance of further SBOs after therapy that exceeded their prior post-surgical experiences. We hypothesize the positive responses from the therapy were due to the reduction of adhesions and the avoidance of new post-surgical adhesions that were causing SBOs and pain in both patients. The use of this non-surgical procedure appears to be of direct patient benefit, and the cost is significantly lower than surgery (SBO surgery costs $114,175; adhesiolysis surgery costs $65,955; this therapy costs $5,200). Unlike abdominal surgery, the therapy has no associated risks from general anesthesia. Data to date (from approximately 50 patients seeking therapy for SBO) shows that it appears to have little or no risk of major adhesion formation, infection or peritonitis. No hospital stay is required with the therapy, because there is virtually no recovery period. 

The use of manual physical therapy is not a new concept in the treatment of patients for a wide variety of medical conditions, including the management of adhesions [21,22,23,24,25,26]. Orthopedic physicians refer to manual physical therapy for treatment of adhesive capsulitis [27]. Oncologists call for manual therapy to treat post-mastectomy scars [28]. Thus, this new utilization for this manual physical therapy regimen has some precedent in clinical care. 

A recently published study suggested that massage therapy reduces inflammation after muscle damage by decreasing the levels of inflammatory cytokines produced [29]. This correlates with our observations that symptoms hypothesized to be attributed to the presence of adhesions do not reoccur after attempted lysis via manual physical therapy. Another study in animals investigated whether a manual massage technique of a surgical area was capable of preventing the formation of adhesions. That study demonstrated fewer adhesions in treated animals as compared to untreated controls [30]. In another study, the manual technique used in these two cases was documented in blinded radiographic reports to open fallopian tubes that were occluded by pelvic adhesions. The long history of clinical physician referrals from other branches of medicine for manual therapy to treat adhesive conditions, along with the studies noted above, support the observation that manual lysis of adhesions may be a viable alternative to SBO surgical treatment.

Without visual confirmation, we can only hypothesize that the manual soft tissue physical therapy protocol effectively lyses the adhesions by causing microfailure of the attachments of the individual crosslinks, allowing return of normal anatomical organization, and formation of more normal tissue structures [31,32,33,34]. If true, these micro-failures of crosslinks and macro-failures of adhesions appear to have similar immediate end results for the patient as surgical adhesiolysis. However, manual manipulation of the soft tissue does not appear to activate the level of inflammation observed with surgical healing. Thus, the adhesions appear to be lysed without the significant induction of new adhesion formation that often follows abdominal and pelvic surgery. 

This hypothesis from our human based case report is supported by historical data in rat models for post-surgical healing in which manual physical therapy performed to soft tissues of the surgical incision site, with animals treated exhibited increased healing as compared to no treatment [35,36]. This therapy also avoids the risks of anesthesia, and the high cost and moderate death rate of surgery. It also appears to minimize the risk of surgical infection, and hospitalization following surgery. Because manual physical therapy has virtually no recovery time, patients are able to recover and participate in daily activities immediately. In follow-up assessments, this return of mobility appears to further deter the reformation of adhesions, and enhance return of more normal tissue organization. This is the same idea that surgeons promote when surgical patients are encouraged to walk and exercise shortly after surgery. Early mobility promotes increased blood flow and an earlier return of normal range of motion. We note that patients who underwent the manual therapy demonstrated increased range of motion in both cases; an early return to normal activities of daily living simply enhanced the benefits.

A formal, well-structured clinical trial is currently underway for the investigation of a nonsurgical approach for the treatment and prevention of SBOs to provide statistical significance to the observations from our human efficacy data presented in this case report.
